# Transference Number in Polymer Electrolytes: Mind the Reference-Frame Gap

**DOI:** 10.1021/jacs.2c02389

**Published:** 2022-04-21

**Authors:** Yunqi Shao, Harish Gudla, Daniel Brandell, Chao Zhang

**Affiliations:** Department of Chemistry−Ångström Laboratory, Uppsala University, Lägerhyddsvägen 1, P.O. Box 538, 75121 Uppsala, Sweden

## Abstract

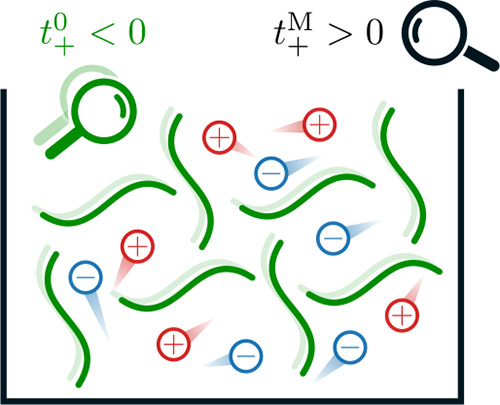

The transport coefficients, in particular the transference number, of electrolyte solutions are important design parameters for electrochemical energy storage devices. The recent observation of negative transference numbers in PEO–LiTFSI under certain conditions has generated much discussion about its molecular origins, by both experimental and theoretical means. However, one overlooked factor in these efforts is the importance of the reference frame (RF). This creates a non-negligible gap when comparing experiment and simulation because the fluxes in the experimental measurements of transport coefficients and in the linear response theory used in the molecular dynamics simulation are defined in different RFs. In this work, we show that, by applying a proper RF transformation, a much improved agreement between experimental and simulation results can be achieved. Moreover, it is revealed that the anion mass and the anion–anion correlation, rather than ion aggregates, play a crucial role for the reported negative transference numbers.

One factor that limits the fast charging and discharging of lithium and lithium-ion batteries is the buildup of a salt concentration gradient in the cell during operation,^1,2^ since the anion flux due to migration must be countered by that of diffusion at steady state. It is therefore desirable for the electrolyte material to carry a greater fraction of cations for migration to minimize the concentration gradient. This fraction, known as the cation transference number, is thus of vital importance in the search for novel electrolyte materials. It is therefore problematic that conventional liquid electrolytes display rather low such numbers and even more troublesome that they are even lower for solid-state polymer electrolytes based on polyethers.

While the condition of a uniform concentration when measuring the transference number can be achieved in typical aqueous electrolytes, its experimental determination in polymer electrolytes is much more challenging due to the continuous growth of the diffusion layer.^3^ At low concentrations, the effect of the concentration gradient may be estimated by assuming an ideal solution without ion–ion interactions, as is done in the Bruce–Vincent method.^4^ At higher concentrations, its effect on the transference number can be taken into account by the concentrated solution theory developed by Newman and can be obtained through a combination of experimental measurements.^5^

The cation transference number  measured in these experiments is defined typically in the solvent-fixed reference frame (RF), denoted by the superscript 0 here.^6^ However, the transference number  as computed in molecular dynamics (MD) simulation based on the linear response theory^7^ is instead related to the velocity correlation functions under the barycentric RF (denoted by the superscript M). This difference creates a conceptual gap when comparing experiments and simulations and interpreting results measured in different types of experiments, when seeking the molecular origin behind the observed phenomenon.

To illustrate this point, we here study a typical polymer electrolyte system: PEO–LiTFSI. For this, a negative  has been reported with Newman’s approach,^8,9^ which has rendered much discussion in the literature.^10−12^ While the formation of ion aggregates has often been suggested to cause such negative ,^11^ only marginally negative values were observed in MD simulations,^13^ even when the correlation due to charged ion clusters was considered explicitly.

To reconcile these observations, we will first investigate how the choice of RF affects the transference number. In fact, it is possible to relate  to  via a simple transformation rule, as shown by Woolf and Harris:^14^
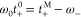
1where the mass fraction of species *i* is denoted as ω_*i*_. According to eq 1, the relation between  and  depends only on the composition, specifically the mass fractions, of the electrolyte.

While the two transference numbers are equivalent at the limit of infinite dilution (ω_0_ → 1), they become distinctly different at higher concentrations. As shown in Figure 1, at the concentration where negative  is observed,  is still positive. Moreover, *t*_+_ generally shifts downward in the solvent-fixed RF as the concentration increases, as seen in Figure 1. This trend can be expected, since at the other limit (ω_0_ → 0),  must converge to the ω_–_ in order to satisfy eq 1. This suggests that  will become increasingly sensitive at higher concentrations since its value will be determined by the motion of a small fraction of solvent molecules. The distinction between  and  may already explain why a negative transference number is seldom observed in MD simulations where the barycentric RF is the default setting. However, more importantly, the strong dependence of *t*_+_ on the RF suggests that the intuitive explanation of the observed negative  being due to the population of ion aggregates is not necessarily the case. Instead, as pointed out in recent studies,^15−19^ the explicit consideration of ion–ion correlations is essential to understand ion transport in polymer electrolytes.

**Figure 1:**
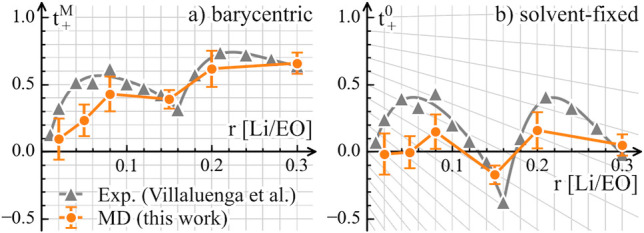
Transference number under (a) barycentric RF and (b) solvent-fixed RF in PEO–LiTFSI for different concentrations *r* [Li/EO] (the ratio of Li to ether oxygen). The conversion rule of *t*_+_ as determined by eq 1 is shown by projecting the grid of part a to part b. The experimental data and fitting of  are reproduced from ref (8). The transfer numbers in MD simulations are computed from the corresponding Onsager coefficients using eq 3; see the Supporting Information for simulation details.

In the following, we will show how the ion–ion correlations contribute to the negative transference number in light of the RF. In the Onsager phenomenological equations,^20^ the flux  of species under a reference frame S can be considered as the linear response of the external driving forces **X**_*j*_ acting on any species *j*:
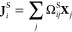
2where  are the Onsager coefficients. For the index *j*, here we denote the solvent as 0, the cation as +, and the anion as −. In addition, the fluxes satisfy the following RF condition: , where  are the proper weighing factors, i.e.,  for the barycentric RF and  for the solvent-fixed RF.^21^ Then, a unique set of the Onsager coefficients can be determined by applying the Onsager reciprocal relation, , and the RF constraint, .

Knowing these Onsager coefficients, one can express the transport properties of interest here, i.e., the transference number and the ionic conductivity, as
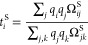
3
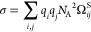
4where *q*_*i*_ is the formal charge of species *i* and *N*_A_ is the Avogadro constant. It is worth noting that, unlike the transference number, the ionic conductivity is RF-independent because of the charge neutrality condition.

While the transformation of *t*_+_ from the solvent-fixed RF to the barycentric RF can follow the straightforward rule of eq 1, the corresponding RF transformation of Ω_*ij*_ is not trivial. This is illustrated by a simplified example shown in Figure 2, where the driving force acting on the cation is assumed to be zero. In the barycentric RF, both driving forces **X**_0_ acting on the solvent and **X**_–_ acting on the anion will contribute to the anion flux . When transforming the Onsager coefficients to the solvent-fixed RF, only the driving force **X**_–_ contributes to the anion flux , as  by construction.

**Figure 2:**
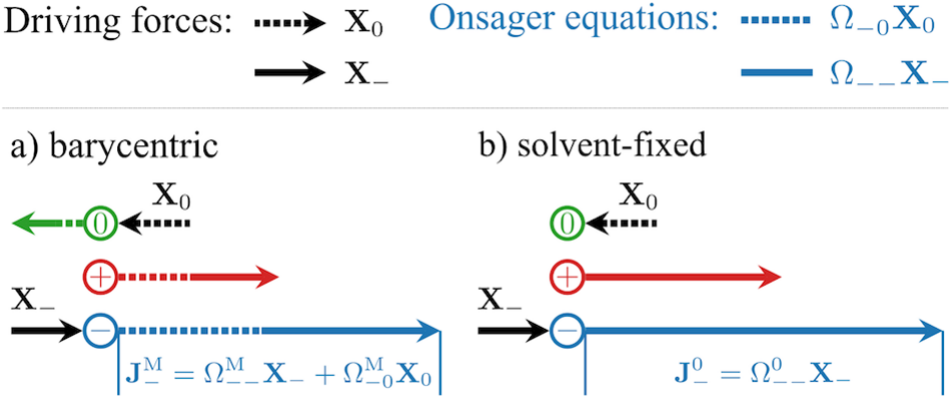
An illustration of the transformation procedure when converting  to  for the case where the driving force acting on the cation is zero. The dashed lines indicate relevant parts related to the solvent.

Nevertheless, the general transformation rule can be derived using the independent fluxes and driving forces,^21^ which is consistent with the above constructions. Following the notation of Miller,^22^ one can consider only the *n* – 1 independent fluxes and driving forces in an *n* component system, where the flux of the solvent **J**_0_ is treated as a redundant variable. This leads to the following set of rules for the RF transformation:
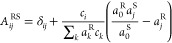
5
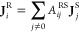
6
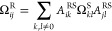
7where  is the matrix that converts the independent fluxes from the reference frame S to R, and *c*_*i*_ is the molar concentration of species *i*. The coefficients  may then be fixed according to the RF constraint. The specific transformation equations for the barycentric and solvent-fixed RFs are provided in the Supporting Information.

This transformation provides the connection between  measured experimentally and  derived from MD simulations. Thus, one can compare Onsager coefficients under a common RF to see whether the simulation describes the same transport mechanism as in experiment or not. Here, we computed Onsager coefficients following Miller’s derivation^6^ with experimental measurements by Villaluenga et al.^8^ MD simulations were performed using GROMACS^23^ and the General AMBER Force Field,^24^ from which Onsager coefficients were derived with in-house analysis software. Details of the conversion and simulation procedure can be found in the Supporting Information. In addition, we shall note here that an alternative set of transport coefficients, i.e., the Maxwell–Stefan diffusion coefficients, were originally reported from experiment,^8^ and they are consistent with the present framework (see the Supporting Information for the interconversion). In addition, the Onsager phenomenological equations may also be written in terms of the resistance coefficients,^25^ which closely resemble the Maxwell–Stefan equations. However, the Onsager coefficients are favored here because they are well-behaved at any given concentration and therefore helpful to understand the RF dependency of the ion–ion correlations.

As shown in Figure 3, the conductivity and Onsager coefficients obtained from MD simulations generally match the experimental values. In particular,  is negative in the entire concentration range, and this indicates an anticorrelation between cations and anions. Furthermore, we see that the experimentally observed negative transference number at *r* = 0.15 is reproduced in the MD simulation, with consistent features of Ω_*ij*_, namely,  and . These results demonstrate that the experimentally observed negative transference number in PEO-LiTFSI systems is captured with the present force field parametrization used in the MD simulations.

**Figure 3:**
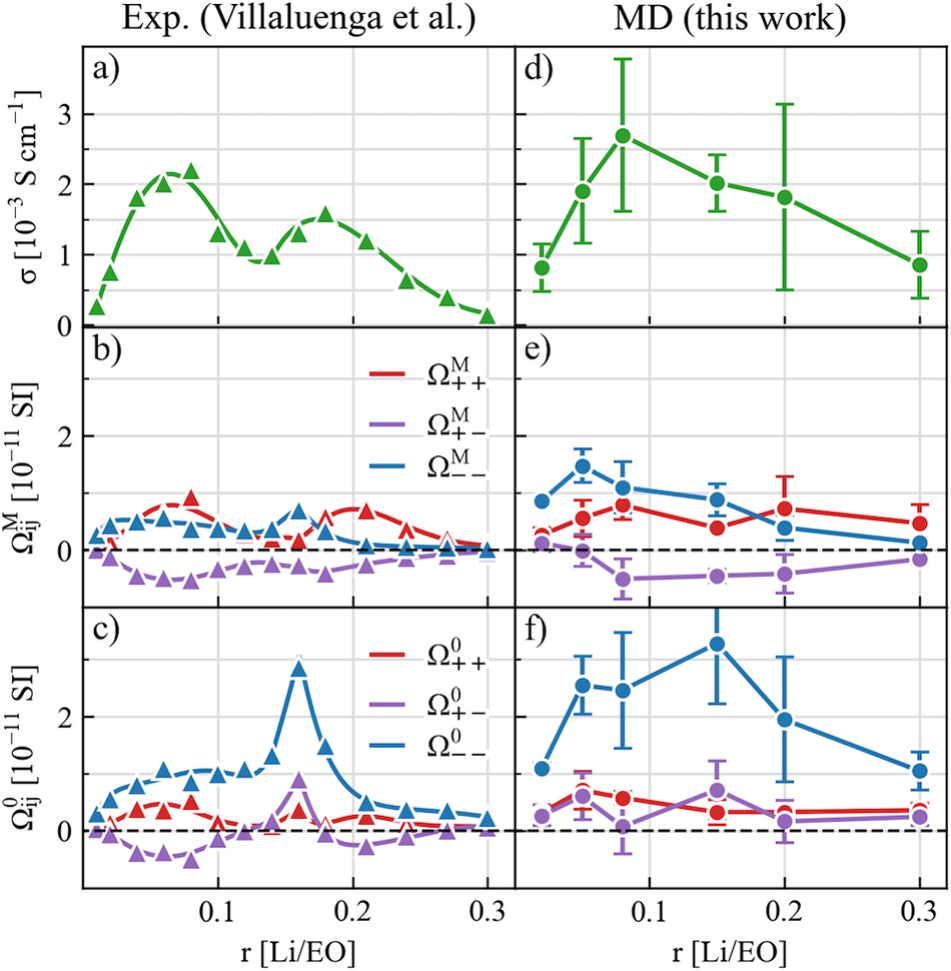
Ionic conductivity and Onsager coefficients under the barycentric and solvent-fixed RF derived from (a–c) experimental measurements and (d–f) MD simulations. The experimental measurements (▲) and fittings (curved lines) are converted from ref (8). The MD simulation results are computed by fitting the mean cross displacements, as detailed in the Supporting Information.

Looking at the effects of RF, we see that Ω_––_ and Ω_+–_ changes more significant upon RF transformation as compared to Ω_++_. In particular, at *r* = 0.15,  is negative while  is positive. This means that the driving force applied to the cations correlates to a codirectional anion flux in the solvent-fixed RF but that an opposite anion flux is found in the barycentric RF. This, together with the observations made above, cannot be explained by any distribution of ideal charge carrying clusters.

To better understand the underlying physical account, we can look into the Onsager coefficients from a microscopic point of view, as they are related to the correlation functions of the fluxes. From the equations shown below, it is clear that the RF transformation is equivalent to transforming either the current-correlation function shown in eq 8 or, equivalently, the displacements of ions shown in eq 9. Thus, this result (eq 10) is consistent with eq 7 and the Wheeler−Newman expression for .^26^
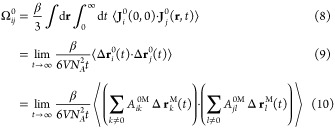
where β = 1/(*k*_B_*T*) is the inverse temperature, and  is the total displacement of species *i* over a time interval *t*.

Based on this result, the conversion of Onsager coefficients upon an RF transformation can be visualized as an affine transformation of ion displacement, as shown in Figure 4. At *r* = 0.15, the displacement of cations and anions is apparently anticorrelated in the barycentric RF, while the correlation becomes positive in the solvent-fixed RF. This can be rationalized, since the motion of anions in the barycentric RF entails the motion of solvent in the opposite direction, giving rise to the enhanced anion motion and the positive cation–anion correlation in the solvent-fixed RF. On the other hand, the motion of cations induces a much less significant effect, as signified by the small distortion along the *x*-axis. This indicates that anions play a significant role for the transference number of Li^+^, not only by its relative motion to the cation.

**Figure 4:**
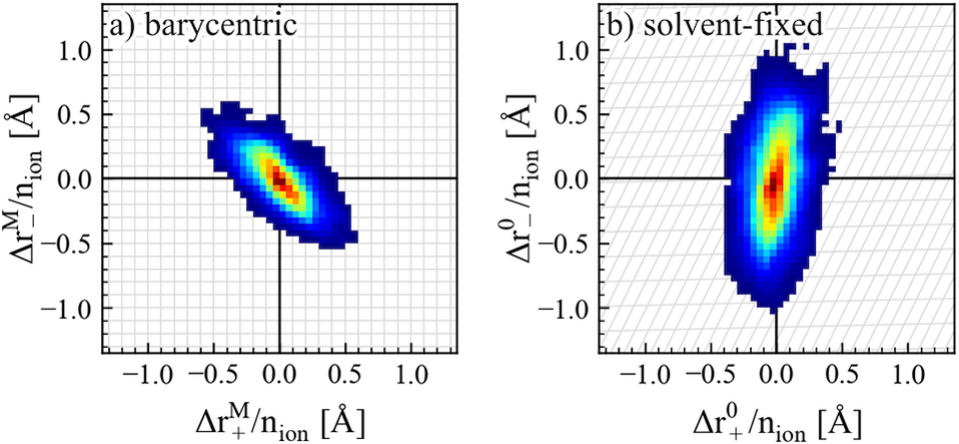
Transformation of the normalized displacement correlations upon a change of reference frame. Δ*r*^M^/*n*_ion_ is the total displacement Δ*r*^M^ (of cations “+” or anions “–”) normalized by the number of ions *n*_ion_. The correlation is obtained from a 400 ns MD trajectory, where the correlation between mean displacements of cations and anions over Δ*t* = 10 ns is plotted in (a) the barycentric RF and (b) the solvent-fixed RF. The RF transformation according to eq 7 is visualized as the projection of grid lines from part a to b.

Indeed, the sign of the experimentally measured  depends not only on , but also on the  and the anion mass fraction. The importance of the anion–anion correlation and the anion mass is demonstrated in Figure 5, where the partial derivative of  shows its strong dependency on the anion mass and Onsager coefficients. An increase of the anion mass introduces an even stronger reduction of the transference number , and therefore,  is more likely to be negative. The same effect occurs when the anion–anion correlation becomes stronger, and  becomes larger. This suggests a direct connection between the observed negative  and a strong anion–anion correlation found at higher concentrations. The latter effect was also indicated in a recent X-ray scattering study of PEO–LiTFSI systems.^12^

**Figure 5:**
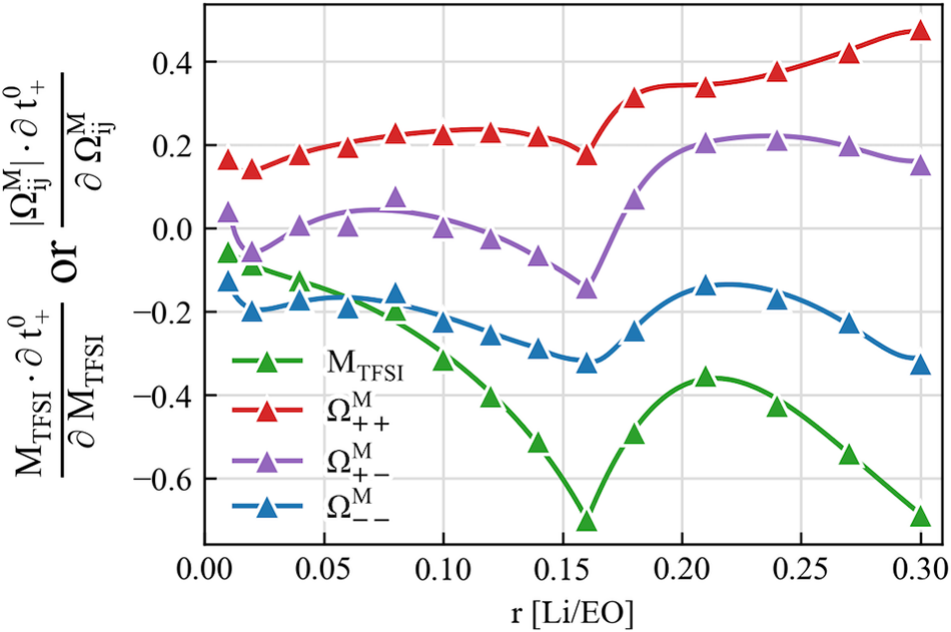
Sensitivity analysis of transference number  in solvent-fixed RF to the variations in the anion molecular weight *M*_TFSI_ and different Onsager coefficients  in the barycentric RF. The analysis is performed by evaluating the partial derivative of  to the logarithm of *M*_TFSI_ or , with data derived from experimental measurements in ref (8). Note that  is mostly negative as shown in Figure 3, while the other variables are positive.

In summary, our present analysis reveals a strong RF dependency of the transference number and the Onsager coefficients in the PEO–LiTFSI system. With a proper transformation, the Onsager coefficients can be used as a rigorous test to compare the transport properties from experimental measurements and MD simulations, as shown here. This will provide new ground to refine force field parametrization, for example, by including the subtle effects of electronic polarization,^27^ although we found that the standard force field already captures the main features observed in experiments.

Not only do our results demonstrate that the experimentally observed negative  can be reproduced with MD simulations, but they also show that cations and anions are mostly anticorrelated in the barycentric RF ( < 0) throughout the entire concentration range in both experiment and simulation. While this does not rule out the possibility of short-lived ion aggregates, neither does it support a transport mechanism based on negatively charged ion clusters. Instead, we show that a large anion mass and strong anion–anion correlations can be responsible for a negative transference number of .

Furthermore, the RF dependence of ion–ion correlations suggests that any discussions about ion–ion correlations need to be had within the same RF. This may shed light on why a different observation was made regarding the sign of *t*_+_ with alternative experimental approaches such as electrophoretic NMR (eNMR).^10^

Although we do not expect that all discrepancies in transport properties between different experimental approaches and between experiment and simulation can be resolved by the present analysis, insights regarding the RF dependency of ion–ion correlations and a direct comparison of the complete set of Onsager coefficients between experiment and simulation as demonstrated in this work would be essential to elucidate the ion transport mechanism in polymer electrolytes and concentrated electrolyte systems alike.

## Supporting Information Available

Details of MD simulations and force field parameters; computation and conversion of Onsager coefficients in different RFs; conversion between different sets of transport equations; and a list of symbols (PDF)
