# Supplementary material for: Transference Number in Polymer Electrolytes: Mind the Reference-Frame Gap
Source: J Am Chem Soc. 2022 Apr 21;144(17):7583–7. doi: 10.1021/jacs.2c02389 (PMC9074101; doi:10.1021/jacs.2c02389)
Supplement: Supplementary file 1 — ja2c02389_si_001.pdf [file ja2c02389_si_001.pdf]

Supporting Information: Transference Number in Polymer Electrolytes: Mind the Reference-frame Gap

Yunqi Shao, Harish Gudla, Daniel Brandell, and Chao Zhang*

Department of Chemistry-Ångström Laboratory, Uppsala University, Lägerhyddsvägen 1, P. O. Box 538, 75121 Uppsala, Sweden

E-mail: chao.zhang@kemi.uu.se

MD simulations and force field parameters

The General AMBER force field (GAFF)[S1] parameters were used for describing bonding and non-bonding interactions in PEO and LiTFSI. The force field parameters along with atomic partial charges were obtained and assigned using ACPYPE[S2] and ANTECHAMBER[S3] tools. These partial charges on the salt were scaled by a factor of 0.75 to effectively introduce electronic polarizations and also previously shown to better reproduce experimental diffusivities.

Table S1: The atomic partial charges of PEO and LiTFSI from GAFF.

PEO		LiTFSI	
Atom type	Charge (e)	Atom type	Charge (e)
C	0.130	C	0.417
O_{chain}	-0.429	S	1.113, 1.261
H_{chain}	0.043	F	-0.204
O_{end}	-0.612	N	-0.794
H_{end}	0.405	O	-0.460, -0.493
		Li	0.750

The initial polymer MD simulation boxes comprising 200 hydroxyl-terminated poly(ethylene oxide) (PEO) chains, each with 25 monomer units (1.11 kg/mol) were constructed using PACKMOL package.[S4] Six different salt concentrations were obtained by adding 100, 250, 400, 750, 1000 and 1500 lithium bis(trifluoromethane)sulfonimide (LiTFSI) molecules, corresponding to a [Li/EO] concentration ratio of 0.02, 0.05, 0.08, 0.15, 0.20, and 0.3, respectively. After the energy minimization step, all systems were equilibrated first with NVT (constant number of particles, volume, and temperature) and then NPT (constant number of particles, pressure, and temperature) runs using the Bussi-Donadio-Parrinello[S5] thermostat and the Parrinello-Rahman barostat[S6] at 400 K and 1 bar with a time step of 1 fs. The thermostat and barostat coupling constants were set to 0.1 and 2.0 ps, respectively. Then, NPT production runs were carried out for additional 400 ns at 430 K to ensure that the Li-ion dynamics has reached diffusive regime and trajectories were saved every 5 ps.

Computation and conversion of Onsager coefficients in different RFs

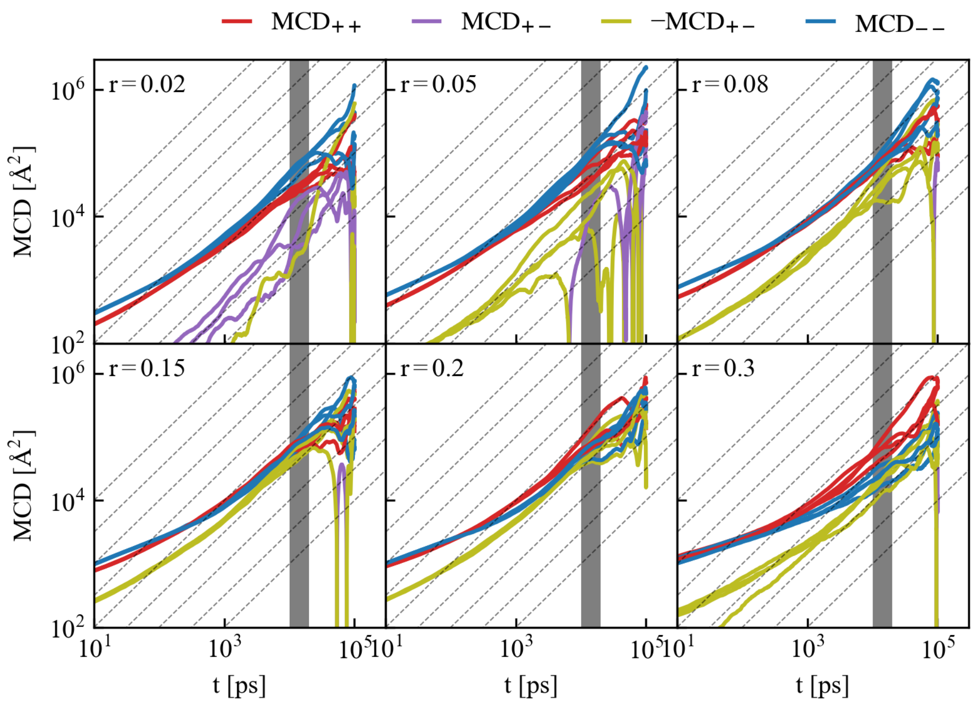

Figure S1: The mean cross displacements (MCDs), defined as $\mathrm{MCD}_{ij} = \left\langle \Delta\mathbf{r}_i^{\mathrm{M}}(t) \cdot \Delta\mathbf{r}_j^{\mathrm{M}}(t) \right\rangle$, as functions of the time interval t at different concentrations. Note that $-\mathrm{MCD}_{+-}$ is also plotted, since cation-anion anti-correlation is observed at higher concentrations. The time window used for the linear fit is shown with gray shading.

The Onsager coefficients from MD simulations are computed from the mean cross displacements (in the barycentric RF):

$$\Omega_{ij}^{\mathrm{M}} = \lim_{t\to\infty} \frac{\beta}{6VN_{\mathrm{A}}^2 t} \left\langle \Delta\mathbf{r}_i^{\mathrm{M}}(t) \cdot \Delta\mathbf{r}_j^{\mathrm{M}}(t) \right\rangle \tag{S1}$$

where β is the inverse temperature, N_{A} is the Avogadro constant and $\Delta\mathbf{r}_i^{\mathrm{M}}(t)$ is the total displacement of species i over a time interval t. The limit is estimated by fitting the correlation $\left\langle \Delta\mathbf{r}_i^{\mathrm{M}}(t) \cdot \Delta\mathbf{r}_j^{\mathrm{M}}(t) \right\rangle$ as a linear function of t over the interval 10-20 ns. At this timescale, all correlation functions reach the diffusive regime, as signified by a slope of 1

in the log-log scale shown in Fig. S1. The MD trajectories were split into segments of 100 ns, and the limit was estimated separately for each segment, where the standard deviation across segments is taken as the error estimation in the main text.

The experimental values of Onsager coefficients for an electrolyte solution of the molar concentration c are computed from the measured conductivity σ, solvent-fixed cation transference number t^0_+, salt diffusion coefficients $D^{\mathrm{V}}_{\mathrm{salt}}$, and the so-called thermodynamic factor $1 + \frac{d \ln \gamma_\pm}{d \ln m}$:

$$\Omega^0_{ij} = \frac{t^0_i t^0_j \sigma}{z_i z_j F^2} + \frac{c D^{\mathrm{V}}_{\mathrm{salt}}}{2RT\left(1 + \frac{d \ln \gamma_\pm}{d \ln m}\right)} \tag{S2}$$

which is equivalent to that derived by Miller.[S7] It is worth noting that $D^{\mathrm{V}}_{\mathrm{salt}}$ is also RF-dependent, and the conversion between different RFs is discussed in Ref. S7. Here, the volume-fixed value is used to be consistent with the result of Balsara and Newman.[S8]

A special form of Eq. 7 in the main text is used to convert the Onsager coefficients for 1:1 electrolytes to solve Ω^{M}_{ij} from Ω^0_{ij} or vice versa. Written as the linear relation between independent sets of Ω_{ij}, one have:

$$\begin{bmatrix} \Omega^0_{++} \\ \Omega^0_{+-} \\ \Omega^0_{--} \end{bmatrix} = \frac{1}{\rho_0^2} \begin{bmatrix} (\rho_0 + \rho_+)^2 & 2\rho_-(\rho_0 + \rho_+) & \rho_-^2 \\ \rho_+(\rho_0 + \rho_+) & \rho_0^2 + \rho_0\rho_+ + \rho_0\rho_- + 2\rho_+\rho_- & \rho_-(\rho_0 + \rho_-) \\ \rho_+^2 & 2\rho_+(\rho_0 + \rho_-) & (\rho_0 + \rho_-)^2 \end{bmatrix} \begin{bmatrix} \Omega^{\mathrm{M}}_{++} \\ \Omega^{\mathrm{M}}_{+-} \\ \Omega^{\mathrm{M}}_{--} \end{bmatrix} \tag{S3}$$

where ρ_0, ρ_+, ρ_- are the mass concentration of the solvent, the cation, and the anion.

Conversion between different sets of transport equations

In the Maxwell-Stefan equations,[S9] the driving force is related to relative velocities between different species through the Maxwell-Stefan diffusion coefficients $\mathfrak{D}_{ij}$ or the friction coefficients K_{ij}:

$$c_i \nabla \mu_i = RT \sum_{j \neq i} \frac{c_i c_j}{c_T \mathfrak{D}_{ij}} (\mathbf{v}_j - \mathbf{v}_i) \tag{S4}$$

$$c_i \nabla \mu_i = \sum_{j \neq i} K_{ij} (\mathbf{v}_j - \mathbf{v}_i) \tag{S5}$$

where $c_T = \sum_i c_i$. It follows that K_{ij} and $\mathfrak{D}_{ij}$ are related as:

$$\frac{RT}{\mathfrak{D}_{ij}} = \frac{c_T}{c_i c_j} K_{ij} \tag{S6}$$

The relation between the Maxwell-Stefan coefficients with the Onsager coefficients can be shown through the modified form of Maxwell-Stefan equations introduced by Newman,[S10] where the relative velocities are replaced with the velocities in the solvent-fixed RF:

$$c_i \nabla \mu_i = \sum_{j \neq 0} M_{ij} (\mathbf{v}_j - \mathbf{v}_0) \tag{S7}$$

M_{ij} are related to K_{ij} as:

$$\begin{bmatrix} K_{+0} \\ K_{+-} \\ K_{-0} \end{bmatrix} = \begin{bmatrix} -1 & -1 & 0 \\ 0 & 1 & 0 \\ 0 & -1 & -1 \end{bmatrix} \begin{bmatrix} M_{++} \\ M_{+-} \\ M_{--} \end{bmatrix} \tag{S8}$$

Inspecting Eq. S7 and the Onsager equations in the solvent-fixed RF (note that $\Omega^0_{i0} = 0$):

$$\mathbf{J}^0_i = c_i (\mathbf{v}_j - \mathbf{v}_0) = -\sum_{j \neq 0} \Omega^0_{ij} \nabla \mu_j \tag{S9}$$

It is clear that the M_{ij} and Ω^0_{ij} are related with the matrix inversion of transport coefficients, for 1:1 electrolyte solution with concentration c:

$$\begin{bmatrix} M_{++} & M_{+-} \\ M_{+-} & M_{--} \end{bmatrix} = -c^2 \begin{bmatrix} \Omega^0_{++} & \Omega^0_{+-} \\ \Omega^0_{+-} & \Omega^0_{--} \end{bmatrix}^{-1} \tag{S10}$$

or:

$$\begin{bmatrix} M_{++} \\ M_{+-} \\ M_{--} \end{bmatrix} = \frac{c^2}{\det(\boldsymbol{\Omega})} \begin{bmatrix} -\Omega^0_{--} \\ \Omega^0_{+-} \\ -\Omega^0_{++} \end{bmatrix} \tag{S11}$$

with:

$$\det(\boldsymbol{\Omega}) = \Omega^0_{++}\Omega^0_{--} - \left(\Omega^0_{+-}\right)^2 \tag{S12}$$

$$= \frac{c\sigma D^{\mathrm{V}}_{\mathrm{salt}}}{2RTF^2\left(1 + \frac{d\ln\gamma_\pm}{d\ln m}\right)} \tag{S13}$$

The consistency between the different sets of transport coefficients may be shown by representing $\mathfrak{D}_{ij}$ in terms of Ω^0_{ij}:

$$\mathfrak{D}_{+0} = \frac{c_0 RT}{c_T c} \cdot \frac{\det(\boldsymbol{\Omega})}{\Omega^0_{--} - \Omega^0_{+-}} \tag{S14}$$

$$\mathfrak{D}_{+-} = \frac{RT}{c_T} \cdot \frac{\det(\boldsymbol{\Omega})}{\Omega^0_{+-}} \tag{S15}$$

$$\mathfrak{D}_{-0} = \frac{c_0 RT}{c_T c} \cdot \frac{\det(\boldsymbol{\Omega})}{\Omega^0_{++} - \Omega^0_{+-}} \tag{S16}$$

Combining Eq. S14-S16 and Eq. S2, we arrived at the same relation between $\mathfrak{D}_{ij}$ and experimental measurables, as is studied in Ref. S8 and S11, which proves the consistency

between different descriptions of transport coefficients.

$$\mathfrak{D}_{+0} = \frac{c_0 D_{\text{salt}}^{\text{V}}}{2c_T t_-^0 \left(1 + \frac{d \ln \gamma_\pm}{d \ln m}\right)} \tag{S17}$$

$$\mathfrak{D}_{+-} = \left[\frac{c_T F^2}{\sigma R T} - \frac{2 t_+^0 t_-^0 \left(1 + \frac{d \ln \gamma_\pm}{d \ln m}\right)}{c D_{\text{salt}}^{\text{V}}}\right]^{-1} \tag{S18}$$

$$\mathfrak{D}_{-0} = \frac{c_0 D_{\text{salt}}^{\text{V}}}{2c_T t_+^0 \left(1 + \frac{d \ln \gamma_\pm}{d \ln m}\right)} \tag{S19}$$

List of symbols

a_i^{S}	weighing factor of species i under reference frame S
A_{ij}^{RS}	matrix elements to convert the independent fluxes from reference frame S to R
c	salt concentration
c_i	molar concentration of species i
c_T	total molar concentration
$D_{\mathrm{salt}}^{\mathrm{V}}$	salt diffusion coefficient under volume-fixed reference frame
$\mathfrak{D}_{ij}$	Maxwell-Stefan diffusion coefficient for species i and j
F	Faraday's constant
$\mathbf{J}_i^{\mathrm{S}}$	flux of species i under reference frame S
k_{B}	Boltzmann constant
K_{ij}	friction coefficient for species i and j
m	molality
M_{ij}	modified friction coefficient for species i and j
M_{i}	molecular weight of species i
n_{ion}	number of ions (cation or anion)
N_{A}	Avogadro constant
q_i	formal charge of species i
r	salt concentration ratio [Li/EO]
$\mathbf{r}_i^{\mathrm{S}}$	summed coordinate vector of species i under reference frame S
R	gas constant
t	time interval
t_i^{S}	transference number of species i under reference frame S
T	temperature
$\mathbf{v}_i$	mean velocity of species i
$\mathbf{X}_i$	external driving force on species i
z_i	charge number of species i

$\gamma_{\pm}$	mean molal activity coefficient of the salt
δ_{ij}	Kronecker delta function
μ_i	mobility of species i
ρ	density
ρ_i	mass concentration of species i
σ	total ionic conductivity
β	inverse temperature
ω_i	mass fraction of species i
Ω_{ij}^{S}	Onsager coefficient for species i and j under reference frame S

References

(S1) Wang, J.; Wolf, R. M.; Caldwell, J. W.; Kollman, P. A.; Case, D. A. Development and Testing of a General Amber Force Field. *J. Comput. Chem.* **2004**, *25*, 1157–1174.

(S2) Sousa Da Silva, A. W.; Vranken, W. F. ACPYPE - AnteChamber PYthon Parser interfacE. *BMC Res. Notes* **2012**, *5*, 367.

(S3) Wang, J.; Wang, W.; Kollman, P. A.; Case, D. A. Automatic Atom Type and Bond Type Perception in Molecular Mechanical Calculations. *J. Mol. Graph. Model.* **2006**, *25*, 247–260.

(S4) Martínez, L.; Andrade, R.; Birgin, E. G.; Martínez, J. M. PACKMOL: A Package for Building Initial Configurations for Molecular Dynamics Simulations. *J. Comput. Chem.* **2009**, *30*, 2157–2164.

(S5) Bussi, G.; Donadio, D.; Parrinello, M. Canonical Sampling through Velocity Rescaling. *J. Chem. Phys.* **2007**, *126*, 014101.

(S6) Parrinello, M.; Rahman, A. Polymorphic Transitions in Single Crystals: A New Molecular Dynamics Method. *J. Appl. Phys.* **1981**, *52*, 7182–7190.

(S7) Miller, D. G. Application of Irreversible Thermodynamics to Electrolyte Solutions. I. Determination of Ionic Transport Coefficients l_{ij} for Isothermal Vector Transport Processes in Binary Electrolyte Systems. *J. Phys. Chem.* **1966**, *70*, 2639–2659.

(S8) Balsara, N. P.; Newman, J. Relationship between Steady-State Current in Symmetric Cells and Transference Number of Electrolytes Comprising Univalent and Multivalent Ions. *J. Electrochem. Soc.* **2015**, *162*, A2720–A2722.

(S9) Krishna, R.; Wesselingh, J. The Maxwell-Stefan Approach to Mass Transfer. *Chem. Eng. Sci.* **1997**, *52*, 861–911.

(S10) Wheeler, D. R.; Newman, J. Molecular Dynamics Simulations of Multicomponent Diffusion. 1. Equilibrium Method. *J. Phys. Chem. B* **2004**, *108*, 18353–18361.

(S11) Villaluenga, I.; Pesko, D. M.; Timachova, K.; Feng, Z.; Newman, J.; Srinivasan, V.; Balsara, N. P. Negative Stefan-Maxwell Diffusion Coefficients and Complete Electrochemical Transport Characterization of Homopolymer and Block Copolymer Electrolytes. *J. Electrochem. Soc.* **2018**, *165*, A2766–A2773.
